# Genetics Influences Drug Consumption in Medication Overuse Headache, Not in Migraine: Evidence From Wolframin His611Arg Polymorphism Analysis

**DOI:** 10.3389/fneur.2020.599517

**Published:** 2021-01-22

**Authors:** Cherubino Di Lorenzo, Giorgio Di Lorenzo, Gianluca Coppola, Vincenzo Parisi, Gaetano S. Grieco, Filippo Maria Santorelli, Esterina Pascale, Francesco Pierelli

**Affiliations:** ^1^Department of Medico-Surgical Sciences and Biotechnologies, Sapienza University of Rome Polo Pontino, Latina, Italy; ^2^Chair of Psychiatry, Department of Systems Medicine, University of Rome Tor Vergata, Rome, Italy; ^3^IRCCS Fondazione Santa Lucia, Rome, Italy; ^4^IRCCS Fondazione G.B. Bietti per lo Studio e la Ricerca in Oftalmologia, Rome, Italy; ^5^Genomic and Post-Genomic Center, IRCCS Fondazione Istituto Neurologico Casimiro Mondino, Pavia, Italy; ^6^Molecular Medicine, IRCCS Fondazione Stella Maris, Calambrone, Italy; ^7^IRCCS Istituto Neurologico Mediterraneo Neuromed, Pozzilli, Italy

**Keywords:** wolframin (WFS1), migraine, medication overuse headache (MOH), pharmacogenomics, single nucleotide polymorphism (SNP)

## Abstract

**Background:** The Wolframin His611Arg polymorphism can influence drug consumption in psychiatric patients with impulsive addictive behavior. This cross-sectional study aims to assess the prevalence of the Wolframin His611Arg polymorphism in MOH, a secondary headache belonging to the spectrum of addictive disorders, episodic migraine (EM), and healthy subjects (HS), and its influence on drug consumption.

**Methods:** One-hundred and seventy-two EM, 107 MOH, and 83 HS were enrolled and genotyped for the Wolframin His611Arg polymorphism. Subjects were classified as homozygous for allele His (H/H subjects), homozygous for allele Arg (R/R subjects), and heterozygous (H/R subjects), regrouped as R/R and carriers of allele H (non-R/R), and matched for clinical data.

**Results:** There were no differences in allelic distributions between the three groups (*p* = 0.19). Drug consumption and other clinical characteristics were not influenced by the Wolframin His611Arg polymorphism (*p* = 0.42; β = 0.04) in the EM group. Among the MOH population, R/R subjects consumed more analgesics (*p* < 0.0001; β = −0.38), particularly combination drugs (*p* = 0.0001; *d* = 2.32).

**Discussion:** The Wolframin His611Arg polymorphism has a similar prevalence between the MOH, EM, and HS groups. The presence of the R/R genotype does not influence symptomatic drug consumption in EM, whereas it determines an increased use of symptomatic drugs in the MOH group, in particular combination drugs (i.e., drugs containing psychoactive compounds).

**Conclusions:** Our findings are consistent with the hypothesis that the Wolframin His611Arg polymorphism plays its effect only in the MOH population, influencing the impulsivity control underlying addictive behavior.

Medication overuse headache (MOH) is a chronic form of secondary headache, usually developed by migraineurs in response to analgesic overuse (1), characterized by a relevant impact in clinical practice, with a prevalence of 0.5–2.6% in the adult population (2). The withdrawal of the symptomatic overuse usually, but not invariably, resolves MOH (1, 3); however, the recurrence into overuse after weaning from drugs is high and unrelated to the modality of detoxification (4) but is influenced by the presence of other forms of abuse (5). Additionally, MOH can be induced in susceptible patients by overuse of analgesics to treat other forms of pain (1) or drugs without a clear analgesic effect to treat another disease that somehow act on migraines (6).

MOH was thought by some authors to belong to the spectrum of addictive disorders (7, 8). According to this hypothesis, the withdrawal syndrome is represented by the chronic headache, the physical dependence is treated by detoxification, the psychological dependence accounts for the recurrences, and the genetic background influences the degree of disease severity by a pharmacogenomics effect (9). To support this theory, our group explored the influence of some genetic backgrounds, already related to substance dependence, on the clinical and physiological characteristics of patients with MOH (10–12). In particular, we analyzed the role of the His611Arg polymorphism of the wolframin gene (WFS1) in a sample of patients with MOH and found that patients homozygous for the rarer genotype (R/R subjects) experienced a more severe form of MOH in terms of doses of drugs consumed and depressive symptoms (10). At the time of our previous study, some authors regarded WFS1 as a promising gene in the development of abuse behavior (13), in particular, because related to two typical personality traits of addicted people: novelty seeking and impulsivity (14). Unfortunately, in the last decade, no other authors addressed studies on WFS1 to the topic, so our hypothesis of WFS1 gene polymorphism contributes to MOH by influencing the impulsivity control underlying addictive behavior, remained unproved.

This study aims to assess the influence of WFS1 His611Arg polymorphism on drug consumption in patients with EM and MOH and detect a possible difference in the prevalence of the analyzed polymorphism among patients and HS. We hypothesized that only in MOH patients the WFS1 His611Arg polymorphism can contribute to the propensity to high symptomatic drug consumption.

## Methods

### Patients

Patients with diagnoses of EM and MOH were screened, between January 2012 and December 2014, at the outpatient Headache-Unit of our Hospital, according to the accepted International Classification of headache disorders (ICHD)-2 criteria. After the release of the ICHD-3 criteria, all patients' files were reanalyzed, and the diagnoses were also confirmed according to these revised criteria. Socio-demographic and clinical data were obtained after an accurate anamnesis performed by a headache-skilled neurologist. At the time of their first visit to the outpatient Headache-Unit, patients were instructed to complete a headache diary to record clinical data (consumption of analgesic drugs per month, days with headache per month, days with drug consumption per month). Inclusion criteria were: (a) the patient's written informed consent; (b) absence of other major medical conditions that potentially could worsen headache or requires a regular pharmacologic treatment (only hormonal treatments, namely pill, patch, and ring, for contraceptive purposes were allowed); (c) absence of psychiatric disorders that could sustain a chronic pain condition (psychotic and/or somatoform disorders); (d) accurate completion of the last 3-month headache diary; (e) diagnostic confirmation of EM or MOH by diary analysis and. Moreover, subjects were screened with the Snellen visual acuity test and with the pure tone audiometry test in order to exclude the visual and auditory deficits related to the Wolfram Syndrome (15, 16). The absence of optic atrophy was assessed by an ophthalmological evaluation including best-corrected visual acuity, slit-lamp biomicroscopy, intraocular pressure measurement, and indirect ophthalmoscopy.

Consecutive outpatients that matched the inclusion criteria were enrolled in the current study at the end of a routine visit. Episodic migraine patients had no history of medication overuse The MOH had ≥15 headache days per month but improved (headache reverts to episodic pattern, <15 headache days per month) after detox and were thus included. Consistent with our previous study (11), the drug types were grouped in four classes: triptans, non-steroidal anti-inflammatory drugs (NSAIDs), associations (i.e., consumption of different types of drugs), combinations (i.e., drugs containing more than one active principle, including psychoactive compounds). The control group was composed by HS of comparable gender distribution as the headache groups, recruited among hospital employees and patients' spouse, that has no headache, neurological, psychiatric and other major medical conditions. All the recruited subjects were independent from those enrolled in the previous study (10). Our Institutional Ethical Committees approved the study.

### Detoxification Protocols

The detoxification protocol depends on each patient. All patients received the advice to stop the use of analgesics for at least 5 weeks (3) and underwent educational training to manage their attacks by antiemetics in case of nausea, myorelaxants in case of neck muscle contraction, and benzodiazepines in case of pain-induced anxiety. Moreover, it was suggested the use of an ice bag to treat the pain if unsupportable. In case of headache worsening, it was allowed the intramuscular injection (IM) of dexamethasone 4 mg, maximum twice per week. Patients with a previous story of multiple medication overuse relapsing received a prescription of prednisone p.o. during the first 10 days (60 mg/day, 2 days; 40 mg/day, 2 days; 20 mg/day, 6 days). The latter group of patients was not allowed to use dexamethasone as rescue medication but was prescribed ketorolac 30 mg IM, maximum twice per week. Finally, patients who overuse drugs containing opiates or butalbital were also treated with decremental therapy with benzodiazepines.

### Molecular Analysis

After obtaining the patients' written informed consent, 10 ml of peripheral blood were collected, total genomic DNA was purified, and patients were genotyped for the WFS1 His611Arg polymorphism by a PCR-RFLP (restriction fragment length polymorphism) method. Briefly, PCR was performed in a total volume of 20 μl consisting of: 40–100 ng of DNA; 200 μM each of dATP, dGTP, dCTP, and dTTP; 10 pmol of the specific primers; and 0.2 U of Taq (GE Healthcare, Cologno Monzese, Italy). A PCR product of 139 bp was obtained using specific primers (Primm, Milan, Italy, www.primm.it): WFS1-F 5′-GAGCTCACCAAGATCGCAGT-3′, and WFS1-R 5′-ACACCAGGATGAGCTTGACC-3′. The PCR reaction consisted of an initial denaturation at 94°C for 5 min, followed by 40 cycles of 30 s of denaturation at 94°C, 30 s of annealing at 59°C, and 30 s of extension at 72°C. A final extension step was performed for 5 min at 72°C. Seven microliters of PCR product were cleaved overnight at 37°C with the endonuclease BsrI (New England Biolabs, Pero, Italy) in a total volume of 10 μl. The cleaved fragments were separated after electrophoresis on a 2.5% agarose gel and stained with ethidium bromide (10).

By these procedures, we detected the presence of the G to A substitution in position 2002 within exon 8 of the gene, accounting for the aminoacidic polymorphism His611Arg (rs734312) (17). According to the results of genetic analysis, patients were classified as homozygous for allele His (H/H subjects), homozygous for allele Arg (R/R subjects), or heterozygous (H/R subjects).

### Power Calculation and Statistical Analysis

Since our primary endpoint was to detect differences among the WFS1 genotype subgroups, the sample size analysis was based on our previous study about the difference in the number of analgesics consumed per month by MOH patients between R/R and carriers of allele H (non-R/R) (10). The monthly drug number consumed by the overall MOH population was 41.82 ± 25.07. The monthly drug number consumed in R/R and non-R/R MOH subgroups was 59.59 ± 31.13 and 37.17 ± 21.15, respectively. To fulfill a desired power of 90% with a significance level at 5% (18), the required sample size was 82 subjects (17 R/R and 65 non-R/R) in the MOH subgroup. Due to the lack of studies about the WFS1 genotype in migraineurs, we decided, as conservative approach, to double the number of subjects (164) required for the migraine sample to detect the difference in number of analgesics consumed per month between R/R and non-R/R with an acceptable power.

All data obtained in the three phases of the study (socio-demographic and clinical recordings and genetic determinations) were merged in a comprehensive database by an independent data-manager, which opened the blind and performed the statistical analyses on definitive data.

Statistics has two levels of analyses: in the first one, there were univariate analyses; in the second one, we carried out two types of multivariate regression models, the multinomial logistic regressions for the categorical dependent variables and the general regression models for continuous dependent variables.

Descriptive statistics to compare WFS1 genotypes were performed among and within diagnostic groups using parametric or, when appropriate, non-parametric tests. *Post hoc* tests were performed with Bonferroni's confidence interval adjustment for multiple comparisons. The Likelihood Ratio Chi-Square test, based on Maximum Likelihood Estimation, was used in both univariate and multivariate models (multinomial logistic regression) with dependent and independent categorical variables. A General Regression Model (GRM) was employed to identify significant predictors of the monthly drug number in the whole headache group and, due to different clinical features of the headache disorders (particularly for the number of analgesics consumed per month), separately in the EM and MOH groups. In addition to well-known indices of null hypothesis significance testing, effect size measures were also reported to recognize the value of the degree of association among variables (19, 20). Effect sizes were calculated with the phi (ϕ) coefficient [or, when appropriate, Cramér's V (ϕ_c_)] for the χ^2^ test and with Cohen's d (d) for Student's *t*-test, with partial Eta squared ($\eta_{p}^{2}$) for analysis of variance (ANOVA). Standardized coefficients (Beta coefficient, β), partial correlations (r_p_), and semi-partial correlations (r_sp_), and GRM effect size estimates were also reported. Statistical significance was set at *p* < 0.05.

## Results

Three-hundred and sixty-two subjects (261 women and 101 men; age, mean ± SD: 42.54 ± 11.52 years) were enrolled in the study: 83 HS (52 women and 31 men; age, 44.57 ± 10.55), 172 EM (125 women and 47 men; age, 39.16 ± 11.14), and 107 MOH patients (84 women and 23 men; age, 46.41 ± 11.36). The three groups and gender were not statistically associated (-2LL = 26.626, $\chi_{2}^{2}\;$ = 5.804, *p* = 0.06). Age was different among three groups (*F*_2,359_ = 15.960, *p* = 0.0001, $\eta_{p}^{2}\;=$ 0.08): migraineurs were younger than controls [*p* = 0.001, d = 0.50 (95% Confidence Interval, CI_95_:−1.77–2.61)] and MOH patients [*p* < 0.0001, d = 0.65 (CI_95_:−1.50–2.31)] whereas controls and MOH did not differ [*p* = 0.77, d = −0.17 (CI_95_: -2.44–1.98)].

The results of the genetic analysis are shown in Table 1. Both the general sample ($\chi_{1}^{2}$ = 1.015; *p* = 0.31) and the three groups separately (HS: $\chi_{1}^{2}\;$ = 0.959, *p* = 0.33; EM: $\chi_{1}^{2}\;$ = 1.128, *p* = 0.29; MOH: $\chi_{1}^{2}\;$ = 0.007, *p* = 0.93) were in Hardy-Weinberg equilibrium.

**Table 1:** Genetic results of polymorphism analysis are reported as frequencies (and percentages) of WFS1 genotypes in HS, EM, and MOH.

	**HS (*n* = 83)**	**EM (*n* = 172)**	**MOH (*n* = 107)**
H/H (*n* = 70)	13 (16%)	28 (16%)	29 (27%)
H/R (*n* = 189)	45 (54%)	91 (53%)	53 (49%)
R/R (*n* = 103)	25 (30%)	53 (31%)	25 (24%)

A preliminary analysis was performed to investigate associations between the three genotypes and the three groups and differences in monthly drug numbers between H/H, H/R, and R/R separately in the EM and MOH groups. The Likelihood Ratio Chi-square test (-2LL = 28.907, $\chi_{4}^{2}\;$ = 6.136, *p* = 0.19) showed that the genotypes were not associated with one of three groups. In the EM patients, the three genotypes did not differ statistically (among them) in the number of monthly used drug (*F*_2,169_ = 1.493, *p* = 0.23, $\eta_{p}^{2}\;$ = 0.02). On the contrary, in MOH patients, there was a difference among the genotypes in the number of monthly used drug (*F*_2,104_ = 10.012, *p* = 0.0001, $\eta_{p}^{2}\;$ = 0.16); R/R patients showed statistically higher values than the H allele carriers (*post hoc* pairwise comparisons: R/R *vs*. H/H, *p* = 0.02; R/R *vs*. H/R, *p* < 0.0001; H/H vs. H/R, *p* = 0.44). These results further confirmed our previous decision (10) to consider together the carriers of allele H in the non-R/R group. To perform further statistical analyses, the general sample was divided into two groups: those with the R/R genotype (103 subjects), homozygotes for allele R, and those with the non-R/R genotype (259 subjects), carriers of allele H, either in heterozygosity or in homozygosity.

A three-way ANOVA (grouping factor: “gender”, “diagnosis”, and “genotype”) showed that age was significantly different among the three diagnostic groups (*F*_2,350_ = 13.544, *p* < 0.0001, $\eta_{p}^{2}$ = 0.07). No further effects (“gender” and “genotype”) or interaction effects (“gender” × “diagnosis”, “gender” × “genotype”, “diagnosis” × “genotype”, and “gender” × “diagnosis” × “genotype”) were significant.

Clinical characteristics and descriptive statistics for the migraine and MOH groups are illustrated in detail in Table 2. In migraineurs, R/R patients had shorter headache durations than non-R/R patients (*p* = 0.002, d = −0.53). In MOH patients, R/R subjects consumed a high number of drugs monthly than the non-R/R patients (*p* < 0.0001, d = 0.97).

**Table 2:** Detailed results of comparisons between WFS1 genotypes in EM group and MOH group.

	**EM group (*****n*** **=** **172)**				**MOH group (*****n*** **=** **107)**			
	**R/R (*n* = 53)**	**non-R/R (*n* = 119)**	**Statistics**	**es**	**R/R (*n* = 25)**	**non-R/R (*n* = 82)**	**Statistics**	**es**
Gender								
Women	38 (72%)	87 (73%)	χ^2^ = 0.04 *p* = 0.85	φ = 0.02	18 (72%)	66 (80%)	χ^2^ = 0.82 *p* = 0.41	φ = 0.09
Men	15 (28%)	32 (27%)			7 (28%)	16 (20%)		
Age	36.94 ± 11.98	40.14 ± 10.65	*t* = −1.75 *p* = 0.8	*d* = −0.29	46.48 ± 10.2	46.39 ± 11.74	*t* = 0.03 *p* = 0.97	*d* = 0.01
Attack frequency^a^	3.53 ± 1.88	3.78 ± 1.43	*t* = −0.88 *p* = 0.38	*d* = −0.16	-	-	-	-
Monthly headache days	6.09 ± 3.43	5.89 ± 3.15	*t* = 0.38 *p* = 0.7	*d* = 0.06	25.32 ± 5.97	25.28 ± 5.93	*t* = 0.03 *p* = 0.98	*d* = 0.01
Headache duration	**20.6** **±** **12.21**	**26.71** **±** **11.47**	***t*** ** = −3.16** ***p*** **=** **0.002**	***d*** ** = −0.53**	31.04 ± 10.09	31.44 ± 13.81	*t* = −0.13 *p* = 0.89	*d* = −0.03
MOH duration	-	-	-	-	5.96 ± 9.3	4.66 ± 5.41	*t* = 0.86 *p* = 0.38	*d* = 0.2
Monthly drug number	6.04 ± 3.04	5.66 ± 2.33	*t* = 0.88 *p* = 0.38	*d* = 0.15	**58.56** **±** **27.35**	**37.93** **±** **19.39**	***t*** **=** **4.21** ***p*** **<** **0.0001**	***d*** **=** **0.97**
Drug type								
Triptans	36 (68%)	71 (60%)	χ^2^ = 1.16 *p* = 0.79	φ_c_ = 0.09	7 (28%)	22 (27%)	χ^2^ = 0.57 *p* = 0.91	φ_c_ = 0.07
Nsaids	7 (13%)	22 (18%)			5 (20%)	13 (16%)		
Associations	4 (8%)	12 (10%)			8 (32%)	26 (32%)		
Combinations	6 (11%)	14 (12%)			5 (20%)	21 (25%)		

*Descriptive statistics [expressed as mean ± SD and frequencies (and percentages)], statistics tests and effect size (es) indices are reported. In bold significant comparisons*.

a *The attack frequency was not reported in MOH group by genotype because MOH is a chronic headache condition often characterized by a continuous of headache days. It makes very difficult to group the headache days in separate attacks*.

### Multivariate Regressions

A full factorial multinomial logistic regression model showed no association between R/R and non-R/R genotypes and the HS, EM, and MOH groups, even after controlling for the “gender” effect and the “genotype” × “gender” interaction effect (-2LL = 35.231, $\chi_{6\;}^{\;2}\;=$ 10.374, *p* = 0.11). When age was inserted in the multinomial logistic regression, the model was significant (-2LL = 441.376, $\chi_{8}^{2}$ = 40.270, *p* < 0.0001). Only age resulted as a predictor of diagnosis (-2LL = 471.273, $\chi_{2}^{2}$ = 29.897, *p* < 0.0001) and particularly predicted the migraine group (B = −0.042, Wald = 11.118, df = 1, *p* = 0.001). This result confirmed no association between genotype and diagnostic group even after controlling for “gender” and age effects.

In the whole headache group, the independent variables entered into the GRM to identify the predictors of monthly drug number (dependent variable) were: age, illness duration, and monthly headache days as continuous variables, and “diagnosis” (EM *vs*. MOH) and “WFS1 genotype” (non-R/R *vs*. R/R) as grouping variables. The model was significant (*F*_6,272_ = 106.384, *p* < 0.0001) and explained 69.5% (adjusted R^2^) of the variance of the monthly drug number. “Diagnosis” (β = −0.49), monthly headache days (β = 0.45), the “diagnosis” × “WFS1 genotype” interaction (β = 0.22), and “WFS1 genotype” (β = −0.20) emerged as significant, independent predictors of pre-detoxification the monthly drug consumption in whole headache group. No further variables considered as possible predictors were entered in the GRM. Detailed results of GRM in whole headache group are reported in upper side of Table 3. The presence of a significant interaction effect between “diagnosis” and “WFS1 genotype” grouping variables on the number of analgesics consumed per month in the whole headache group supported our statistical plan of analyzing headache patients as separated subgroups (EM and MOH) in order to investigate independent predictors of monthly drug number by two different GRMs, one for EM group and one MOH group.

**Table 3:** Detailed results of three General Regression Models (GRMs).

	***F***	**df**	***p***	**$\eta_{p}^{2}$**	**op**	**β (CI_**95**_)**	**r_**p**_**	**r_**sp**_**
Whole headache group								
**Diagnosis**	**36.19**	**1.272**	**<0.0001**	**0.12**	**1.00**	**-0.49 (-0.65-−0.33)**^**a**^	**−0.34**^**a**^	**−0.20**^**a**^
**Monthly headache days**	**32.62**	**1.272**	**<0.0001**	**0.11**	**1.00**	**0.45 (0.29–0.60)**	**0.33**	**0.19**
**Diagnosis** **×** **WFS1 genotype**	**31.43**	**1.272**	**<0.0001**	**0.10**	**1.00**	**0.22 (0.14–0.30)**^**a**^	**0.32**^**a**^	**0.19**^**a**^
**WFS1 genotype**	**33.43**	**1.272**	**<0.0001**	**0.11**	**1.00**	**-0.20 (-0.27–−0.14)**^**a**^	**−0.33**^**a**^	**−0.19**^**a**^
Headache duration	0.04	1.272	0.84	0.00	0.06	0.01 (-0.11–0.14)	0.01	0.01
Age	0.01	1.272	0.93	0.00	0.05	0.01 (-0.12–0.13)	0.01	0.00
EM group								
**Monthly headache days**	**159.73**	**1.160**	**<0.0001**	**0.50**	**1.00**	**0.69 (0.58–0.80)**	**0.71**	**0.45**
**Headache duration**	**8.65**	**1.160**	**0.004**	**0.05**	**0.83**	**-0.26 (-0.44–−0.09)**	**-0.23**	**-0.11**
**Attack frequency**	**19.58**	**1.160**	**<0.0001**	**0.11**	**0.99**	**0.23 (0.13–0.34)**	**0.33**	**0.16**
**Age**	**7.22**	**1.160**	**0.008**	**0.04**	**0.76**	**0.23 (0.06–0.41)**	**0.21**	**0.10**
Drug type × WFS1 genotype	1.81	3.160	0.15	0.03	0.46	−0.11 (-0.22–0.00)^b^	−0.16^b^	−0.07^b^
Drug type	0.69	3.160	0.56	0.01	0.19	0.06 (-0.03–0.15)^b^	0.10^b^	0.05^b^
WFS1 genotype	0.65	1.160	0.42	0.00	0.13	0.04 (-0.06–0.14)^b^	0.06^b^	0.03^b^
MOH group								
**WFS1 genotype**	**19.71**	**1.95**	**<0.0001**	**0.17**	**0.99**	**-0.38 (-0.56-−0.21)**^**c**^	**-0.42**^**c**^	**-0.37**^**c**^
**Drug type** **×** **WFS1 genotype**	**3.94**	**3.95**	**0.01**	**0.11**	**0.82**	**0.29 (0.05–0.53)**^**c**^	**0.24**^**c**^	**0.20**^**c**^
**Monthly headache days**	**9.41**	**1.95**	**0.003**	**0.09**	**0.86**	**0.27 (0.10–0.45)**	**0.30**	**0.26**
Drug type	2.54	3.95	*0.06*	0.07	0.61	−0.27 (-0.52-−0.03)^c^	−0.20^c^	−0.19^c^
MOH duration	1.07	1.95	0.30	0.01	0.18	0.09 (-0.09–0.27)	0.11	0.09
Age	0.08	1.95	0.78	0.00	0.06	0.04 (-0.22–0.29)	0.03	0.02
Headache duration	0.02	1.95	0.89	0.00	0.05	0.02 (-0.23–0.27)	0.01	0.01

*In bold the parameters of three GRM equations are significant independent predictors of monthly drug number, respectively in whole headache group (EM and MOH), EM group and MOH group. Parameters are sorted based on descending β values. df = degree of freedom; $\eta_{p}^{2}$ = partial eta-squared; op = observed power (alpha = 0.05); β (CI_95_) = Beta (95% Confidence Interval); r_p_ = partial correlation; r_sp_ = semi-partial correlation*.

a Effect level with the lowest p value among parameter estimates of whole headache group GRM: “diagnosis”, EM vs. MOH; “WFS1 genotype”, non-R/R vs. R/R; “diagnosis” × “WFS1 genotype”, EM × non-R/R vs. MOH—R/R.

b *Effect level with the lowest p value among parameter estimates of EM group GRM: “drug type”, triptans vs. combinations; “WFS1 genotype”, non-R/R vs. R/R; “drug type” × “WFS1 genotype”, triptans—non-R/R vs. combinations — R/R*.

c *Effect level with the lowest p value among parameter estimates of MOH group GRM: “drug type”, triptans vs. combinations; “WFS1 genotype”, non-R/R vs. R/R; “drug type” × “WFS1 genotype”, triptans—non-R/R vs. combinations—R/R*.

**Figure 1:**
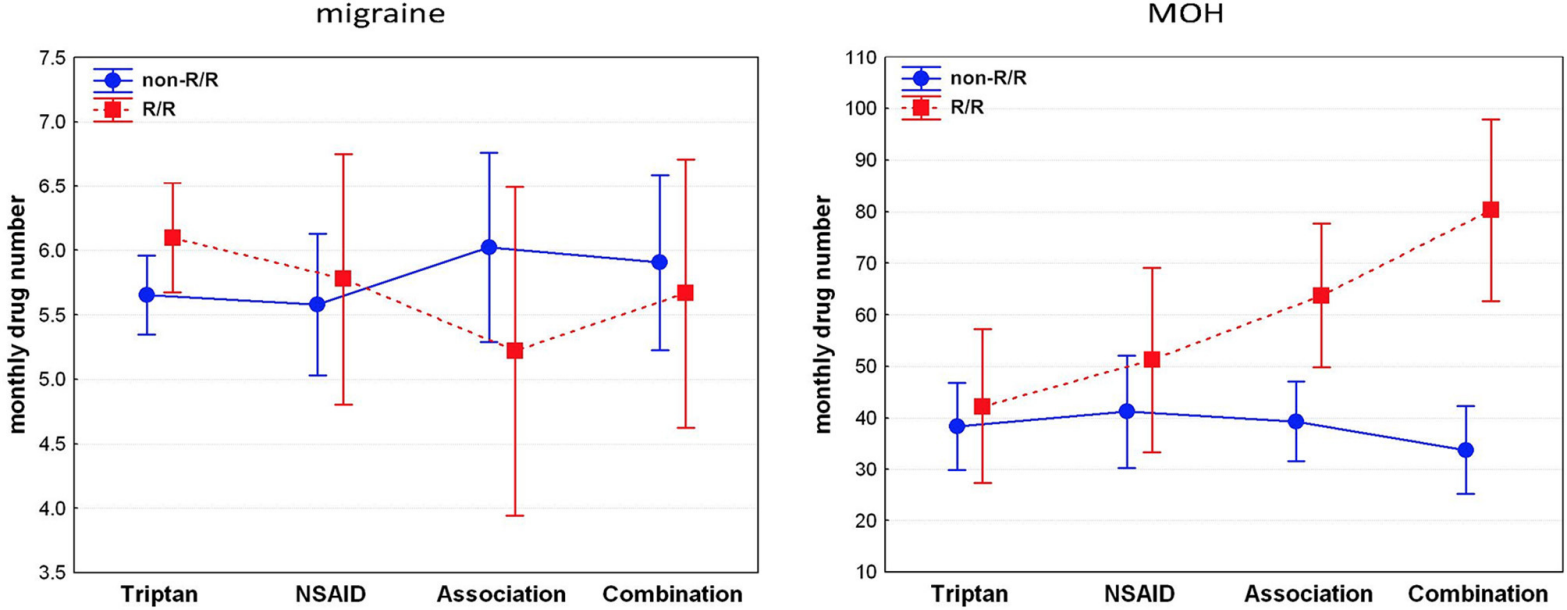
R/R genotype is related to higher consumption of analgesics and preference for combination drugs in patients with MOH, not in migraineurs. NSAIDs: non-steroidal anti-inflammatory drug; associations (i.e., consumption of different types of drugs); combinations (i.e., drugs containing more than one active principle, including psychoactive compounds).

In the EM, the independent variables entered into the GRM to identify the predictors of monthly drug number (dependent variable) were: age, illness duration, headache attack frequency, and monthly headache days as continuous variables, and “WFS1 genotype” (non-R/R *vs*. R/R) and “drug type” (triptans vs. NSAIDs vs. associations vs. combinations) as grouping variables. The model was significant (*F*_11,160_ = 57.211, *p* < 0.0001) and explained 78.3% (adjusted R^2^) of the variance of the monthly drug number. Monthly headache days (β = 0.69), illness duration (β = −0.26), headache attack frequency (β = 0.23), and age (β = 0.23) emerged as significant, independent predictors of the pre-detoxification monthly drug consumption in migraineurs. No further variables considered as possible predictors were entered in the GRM. Detailed results of GRM in EM group are reported in middle of Table 3. The graph in the left panel of the Figure 1 illustrates the number of analgesics consumed per month in EM, separately for the four analgesic classes in both WFS1 genotypes.

In the MOH group, the independent variables entered into the GRM to identify the predictors of monthly drug number (dependent variable) were: age, illness duration, MOH duration, and monthly headache days as continuous variables, and “WFS1 genotype” (non-R/R vs. R/R) and “drug type” (triptans vs. NSAIDs vs. associations vs. combinations) as grouping variables. The model was significant (*F*_11,95_ = 4.303, *p* < 0.0001) and explained 25.5% (adjusted R^2^) of the variance of the monthly drug number. The “WFS1 genotype” (β = −0.38), “drug type” × “WFS1 genotype” interaction (β = 0.29), and monthly headache days (β = 0.27) emerged as significant, independent predictors of pre-detoxification monthly drug consumption in MOH patients. No further variables considered as possible predictors were entered in the GRM. Detailed results of GRM in MOH group are reported in lower side of Table 3. The graph in the right panel of the Figure 1 illustrates the number of analgesics consumed per month in MOH, separately for the four analgesic classes in both WFS1 genotypes. In the *post hoc* analysis, pairwise comparisons of MOH patients that consumed drugs of combination revealed a statistically higher consumption of drug in the R/R group than the non-R/R group [82 ± 33.01 vs. 33.95 ± 18.45; *p* = 0.0001; d = 2.32 (CI_95_:−26.62–10.21)]. Among those that used an association of drugs, the monthly consumption was higher in the R/R group than the non-R/R group; however, this difference did not reach the statistical threshold [63.25 ± 32.80 vs. 38.81 ± 16.84; *p* = 0.09; d = 1.18 (CI_95_:−21.55–7.65)]. Using a less conservative approach with the Fisher's Least Square Difference (LSD) test and a significance threshold adjustment for multiple comparison tests with Bonferroni's correction (0.05/8 = 0.00625), the significance threshold was reached (*p* = 0.003)].

## Discussion

The findings of this study confirm, in a larger sample, our previous result that, within the MOH population, R/R patients have an increased use of symptomatic drugs. In particular, herein we showed that the drug consumption was higher in R/R patients who overuse combination drugs (i.e., drugs containing psychoactive compounds such as caffeine, opiates, or butalbital). According to the study hypothesis, we did not observe the same effect in patients with EM. Moreover, the prevalence of the R/R genotype did not differ among the three examined groups (HS, EM, and MOH).

Wolframin is a membrane calmodulin-binding glycoprotein that resides in the endoplasmic reticulum and regulates cellular Ca^++^ homeostasis (21). In the brain, wolframin is mainly expressed by the limbic system and structures closely related to it (22) and the visual system (retina, optic nerve, brain) (23). Its localization could explain the presence of psychiatric features in Wolfram Syndrome (early-onset diabetes mellitus, progressive optic atrophy, diabetes insipidus, deafness, and psychiatric disorders) and the modulation of WFS1 gene expression in psychiatric, behavioral, and emotional features. As an example, there is evidence that wolframin is synthesized in the amygdala as a consequence of exposure to danger and could be involved in bioactive peptide production (24). A deregulation of these mechanisms is also involved in impaired function of the dopaminergic system (25) and a reduced expression of the alpha1 and alpha2 subunits of GABA(A) receptors (26). As consequence, carriers of dysfunctional WFS1 gene expression could express increased anxiety (26), impaired behavioral adaptation in stressful environments (27), post-traumatic stress disorder (28), and mood disorders (29).

Consistent with our prior papers, we considered the number of analgesic doses consumed monthly by patients as one of the most useful markers of MOH severity in order to stratify patients according to the degree of their likelihood of abuse behavior. In fact, in MOH, the headache frequency ranged from 15 to 30 days per month; on the contrary, the variability of monthly drug consumption ranged from 10 to a not existing hypothetical upper limit. Therefore, according to their drug consumption, patients could be distributed on a wider range that better reflects their disease severity.

In patients with MOH, higher drug consumption would reflect the higher severity of headache or the higher proneness to abuse (i.e., to have poor impulse control). In the previous study, we supposed that the influence of wolframin on MOH is mainly due to an impulsivity-related increased need for drugs, and not to a worsening influence on pain or other primary headache symptoms (10). Interestingly, in the present study all MOH patients reverted to an episodic headache pattern after the detoxification. This point is especially important because it implies that patients' chronic headache was due to medication overuse. So that, we were not dealing with patients affected by a pure chronic migraine (CM). The actual results seem to confirm our earlier hypothesis (10): the MOH clinical picture observed in our sample is an abuse-related disorder and not a simply chronic picture of migraine.

Even if the current headache classification takes together under the diagnosis of MOH (1) both patients who improve and those who do not improve with the detoxification protocols, we are probably facing two different clinical pictures (30). It would be interesting, in the future, to evaluate the role of WFS1 in MOH patients who remain chronic even after detoxification.

In patients with EM, only age and some headache characteristics related to the burden of disease (illness duration, headache attack frequency, and monthly headache days), not the WFS1 genotype, influenced drug consumption. We read this data as the proof of the lack of any effect of the examined polymorphism on headache characteristics and drug consumption behavior in EM. Thereby, the worsening effect of WFS1 genotype we observed only in MOH patients should be interpreted only in terms of influence not on migraine clinical features but on the severity of abuse behavior, exclusively expressed in MOH.

Consistently with our previous hypothesis (10), although no studies analyzed addictive behavior development and drug dependence related to WFS1 gene expression, we suppose that a partial dysfunction of wolframin is predictable in R/R subjects and could sustain the mechanism of addiction observed in our patients. In fact, wolframin could account for the substance dependence because of the limbic structures in which it is expressed, its aminergic and calcium modulation, and its influence on psychiatric, behavioral, and emotional features (31–35). Since it is already known that gene polymorphisms modulate neural plasticity (12, 36, 37), we hypothesize that WFS1 polymorphism could induce synaptic plastic modifications underpinning the development of medication overuse behavior by its modulation of the dopaminergic striatal pathway and intracellular calcium signaling (38–41). Interestingly, alterations of synaptic plasticity were observed in MOH patients, but not in patients with CM, even if they have similar clinical features, excluded the medication overuse that is absent in CM (30, 42). We argue that the examined WFS1 polymorphism may induce a more severe picture of abnormal synaptic plasticity, related to a more severe clinical picture in terms of higher number of symptomatic drug consumption, as we observed in our patients.

Since the effect we observed is higher in patients with MOH who overuse combination drugs, an alternative explanation is that the WFS1 His611Arg polymorphism somehow influences the drug-induced cortical anomalies observed in patients with MOH that are related to the class of symptomatic drugs. In fact, it is well known that the different classes of overused drugs influence the cortical activity of patients with MOH in different ways (43, 44); in patients with MOH, the higher these abnormalities, the higher the drug consumption (44). It is possible to speculate that WFS1 His611Arg could induce the anomalies of cortical activity related to medication overuse and that it results in patients' increased need for drug consumption.

Moreover, we found that the allelic distribution was similar among groups, allowing us to speculate about the possible mechanisms with which WFS1 His611Arg acts in MOH patients. Our results suggest that the polymorphism is not a predisposing factor to development of EM or MOH, i.e., the worsening effect observed among patients with MOH seems to be exerted only after the development of the secondary form of chronic headache due to the medication overuse. It is plausible that, in terms of headache worsening, the genetic polymorphism is silent in EM. When medication overuse leads to the development of MOH, the polymorphism starts to express its worsening effect only because of its potential to affect the abuse behavior (that worsens MOH but does not worsen EM), plausibly by a pharmacogenomics effect (9). Although the genomic aspects of headache have been widely studied for many years (45), pharmacogenomics is poorly explored in headache medicine, even if it is regarded as a promising field of research in various diseases (46). Our view on these results about the entity of medication overuse as a genetically influenced marker of MOH gravity is consistent to another study on abuse behavior in which a genetic background did not lead to the development of the heroin addiction, but only to its higher severity (47).

Finally, certain limitations of the present study should be acknowledged. First, we lack a measure of gene expression to assess if the type of overused drug, detoxification, or His611Arg polymorphism influence WFS1 expression. The eventuality of a drug (or detoxification)-induced epigenetic modification of the observed pharmacogenomics phenomena would result in a very interesting “epipharmacogenomics” effect. Another shortcoming of our study is the absence of a follow-up phase for the analysis of the recurrence of MOH, to assess if the examined polymorphism would result in a higher MOH relapse rate. Unfortunately, the perspective design of the study, the use of different kinds of prophylactic treatments after detoxification, and the long time required to have a recurrence of MOH do not allow the possibility of including the relapse rate in the present study. We plan to pursue this type of observation in further studies.

In summary, because the WFS1 His611Arg polymorphism seems to be unable to influence the development and clinical features of migraine, we suppose that the observed worsening of MOH due to the polymorphism is likely driven by the R/R genotype-related proneness to abuse and, in turn, that the monthly drug consumption in MOH patients is not necessarily the only expression of headache severity. In fact, proneness to abuse is non-influential on EM clinical picture (development and severity) but can worsen MOH. In other terms, since drug consumption is the hallmark of MOH, this form of chronic headache is not a simple worsening of a preexisting migraine but a complex syndrome in which the development of an addictive behavior disorder in a headache patient induces a new clinical picture, sustained by a specific background not shared with EM. We can suppose that drug-induced effects, in joint to a specific genetic background, neuroadaptation, and environmental factors, contribute to the complex mechanisms leading to the development of MOH, despite the WFS1 genotype that, though it may seem strange, stops to be silent only after the start of medication overuse.

## Data Availability Statement

The raw data supporting the conclusions of this article will be made available by the authors, without undue reservation.

## Ethics Statement

The studies involving human participants were reviewed and approved by Institutional Ethics Committee. The patients/participants provided their written informed consent to participate in this study.

## Author Contributions

CDL designed the study and participated to the manuscript draft. GDL performed the statistical analysis and participated to the manuscript draft. GC performed the clinical data recruitment and participated to the manuscript draft. VP performed the ophthalmologic evaluations, aided in interpreting the results, and worked on the manuscript. GG performed the molecular analysis of samples. FS performed the molecular analysis of samples. EP performed the molecular analysis of samples. FP supervised the work and revised the final version of the manuscript. All authors discussed the results and commented on the manuscript.

## Conflict of Interest

The authors declare that the research was conducted in the absence of any commercial or financial relationships that could be construed as a potential conflict of interest.
